# Reliability and validity of Arabic Rapid Estimate of Adult Literacy in Dentistry (AREALD-30) in Saudi Arabia

**DOI:** 10.1186/1472-6831-14-120

**Published:** 2014-09-29

**Authors:** Santosh Kumar Tadakamadla, Mir Faeq Ali Quadri, Amir H Pakpour, Abdulaziz M Zailai, Mohammed E Sayed, Mohammed Mashyakhy, Aadil S Inamdar, Jyothi Tadakamadla

**Affiliations:** Population & Social Health Research Group, Griffith Health Institute, School of Dentistry and Oral health, Griffith University, Gold Coast, Australia; Department of Preventive Dentistry, Faculty of Dentistry, Jazan University, P.O Box: 114, Jazan, 45142 Saudi Arabia; Department of Public Health, Qazvin University of Medical Sciences, Qazvin, Iran; Head, Saudi Dental Society, Ministry of Health, Jazan, Saudi Arabia; School of health related professions, Rutgers University, New Jersey, USA; Department of Endodontics, Jazan University, Jazan, Saudi Arabia

**Keywords:** REALD-30, Arabic, Health Literacy, Dental, Word recognition instrument

## Abstract

**Background:**

To evaluate the reliability and validity of Arabic Rapid Estimate of Adult Literacy in Dentistry (AREALD-30) in Saudi Arabia.

**Methods:**

A convenience sample of 200 subjects was approached, of which 177 agreed to participate giving a response rate of 88.5%. Rapid Estimate of Adult Literacy in Dentistry (REALD-99), was translated into Arabic to prepare the longer and shorter versions of Arabic Rapid Estimate of Adult Literacy in Dentistry (AREALD-99 and AREALD-30). Each participant was provided with AREALD-99 which also includes words from AREALD-30. A questionnaire containing socio-behavioral information and Arabic Oral Health Impact Profile (A-OHIP-14) was also administered. Reliability of the AREALD-30 was assessed by re-administering it to 20 subjects after two weeks. Convergent and predictive validity of AREALD-30 was evaluated by its correlations with AREALD-99 and self-perceived oral health status, dental visiting habits and A-OHIP-14 respectively. Discriminant validity was assessed in relation to the educational level while construct validity was evaluated by confirmatory factor analysis (CFA).

**Results:**

Reliability of AREALD-30 was excellent with intraclass correlation coefficient of 0.99. It exhibited good convergent and discriminant validity but poor predictive validity. CFA showed presence of two factors and infit mean-square statistics for AREALD-30 were all within the desired range of 0.50 - 2.0 in Rasch analysis.

**Conclusions:**

AREALD-30 showed excellent reliability, good convergent and concurrent validity, but failed to predict the differences between the subjects categorized based on their oral health outcomes.

## Background

The twenty first century requires an individual to possess sufficient health related literacy skills, so that one can understand and implement the knowledge or instructions provided by a health care worker [1]. Oral health literacy is defined as “degree to which individuals have the capacity to obtain, process and understand basic oral health information and services needed to make appropriate health decisions” [2]. A survey conducted recently in the United Kingdom found that one out of every five individuals lack the basic skills needed to understand simple information that would help them lead a healthy life [3]. The development seen in psychometrics in the last ten years has helped researchers to use various methods for assessing the health literacy levels among adults. Currently the general health literacy measuring tools include, Rapid Estimate of Adult Literacy in Medicine (REALM) [4], Test of Functional Health Literacy in Adults (TOFHLA), Health Activities Literacy Scale (HALS) and a few others [5].

Oral health being part of general health also requires sufficient attention in terms of measuring and improving the dental literacy skills of the community. Up until the Rapid Estimate of Adult Literacy in Dentistry (REALD-30) was developed by Lee and her colleagues, there was no method available to assess dental literacy in adults [6, 7]. Currently apart from REALD, the other dental literacy instruments available are Test of Functional Health Literacy in Dentistry (TOFHLiD) [8], Oral Health Literacy Instrument (OHLI) [9], Comprehensive Measure of Oral Health Knowledge (CMOHK) [10] and the brief 20-item dental/medical health literacy screen (REALMD-20) [11]. REALD is simple and easy to administer when compared to other oral health literacy instruments. Among these instruments, CMOHK mostly focuses on the knowledge oriented questions, while TOFHLiD and OHLI have Medicaid rights and responsibilities in their content, making them invalid for countries which lack Medicaid facilities. The REALD was made and modeled after REALM and the words were taken from American Dental Association (ADA) Glossary of Common Dental Terminology. At first, this instrument was developed as REALD-30 by Lee et al and it consisted of thirty commonly used dental terminologies [7]. Later another sixty nine words were added to make a longer set, REALD-99, only to cover a wide range of terminologies [6, 7]. The words were incorporated in the increasing order of difficulty and the overall score was obtained by adding the total, giving one point for each word pronounced correctly. The REALD-30 has been tested for reliability and validity to show its effectiveness in measuring the dental health literacy among adults [7, 12, 13]. But the portrayed positive characteristics in determining the literacy levels are limited to specific populations. Use of advancement in the psychometric analysis can provide advantage in testing the instrument in culturally different populations. For researchers to know the literacy levels of a population it is practically necessary to develop an instrument in their native language. This will help in implementing strategies in order to improve the level of understanding and communication between the patients and the health care providers.

In recent years, an increased focus on the improvement of oral health has been observed in most of the Arabic speaking nations such as Saudi Arabia [14]. Considering the importance of oral health literacy for better oral health status, it is important to be equipped with a valid tool for measuring the dental health literacy in the region’s native language. There are approximately 25 nations with nearly 200 million Arabic speaking people [15] in the gulf peninsula and till date there is no tool developed in Arabic language to assess dental health literacy. The objective of this study was to evaluate the reliability and validity of Arabic Rapid Estimate of Adult Literacy in Dentistry (AREALD-30) in Saudi Arabia.

## Methods

### Study population

The target population for the present study constituted patients visiting the outpatient department of dental clinics at College of Dentistry, Jazan University. Patients who fulfilled the inclusion criteria (literate and aged over 25 years) were invited to participate and those who provided the consent comprised the final sample. A total of 200 subjects were invited, of which 177 agreed to participate giving a response rate of 88.5%. A convenience sample of 20 patients was recalled after two weeks for reliability assessment. Ethical approval was obtained from the Ethics committee of Jazan University, Saudi Arabia.

### Arabic translations

A pool of “dentistry related words” was constructed by translating English REALD-99 [6] words into Arabic. World Health Organization (WHO) guidelines for translation and adaptation of instruments were followed [16]. Along with the REALD-99, 14 item Oral Health Impact Profile (OHIP-14) [17] was translated and the Arabic version of REALD-99 (AREALD-99), AREALD-30 and A-OHIP-14 were obtained. Two bilingual dental professionals with Arabic as their native language independently translated English REALD-99 words and OHIP-14 into Arabic. Translators were instructed to aim at the conceptual equivalence of the words but not the literal translation. An expert panel was convened with three bilingual individuals (two of them were dental professionals) to resolve the discrepancies between the independently translated versions. In addition, an independent professional translator back-translated the Arabic version into English and no discrepancies existed between the original and back-translated English versions of AREALD-99 and A-OHIP-14. AREALD-99 and A-OHIP-14 questionnaire were pilot tested on a convenience sample of twenty patients visiting the dental clinics to assess face and content validity. The participants were queried about the difficulties in understanding the items and any changes required were done accordingly.

### Instruments used

Structured interviews were conducted by two bilingual interviewers. Each participant was provided with the list of words mentioned in AREALD-99 [6], which also included the words from AREALD-30 and was asked to read them aloud. Each immediate correct pronunciation for the word received 1 mark, while pauses, hesitations and repetitions received a 0 mark. The total score for AREALD-30 and AREALD-99 thus ranged from 0 to 30 and 0 to 99 respectively (higher total score suggests higher dental literacy level). Other background characteristics recorded were socio-behavioral information like age, gender, education level, dental visiting pattern and self-perceived dental health status (recorded on a five point Likert scale: excellent, very good, good, fair and poor). In addition, A-OHIP-14 was administered. OHIP-14 is a self-administered questionnaire that measures quality of life using 14 items in seven dimensions: functional limitation, physical pain, psychological discomfort, physical disability, psychological disability, social disability, and handicap. Each dimension is measured by two questions [17]. The overall OHIP-14 score for every individual is calculated by summing up scores of each item, higher OHIP-14 scores suggest poorer oral health related quality of life. Similar scoring methodology was adopted to score A-OHIP-14.

### Statistical analysis

To investigate the reliability of the AREALD-30, internal consistency and test-retest reliability were computed. Internal consistency was evaluated using Cronbach α coefficient. AREALD-30 was expected to be internally consistent if it acquired an α coefficient of at least 0.70 [18]. To assess stability of AREALD-30 across times, a test-retest reliability analysis was carried out and the intra-class correlation coefficients (ICC) were computed (ICC agreements; <0.40-poor to fair, 0.41-0.60-moderate, 0.61-0.80-good, >0.80-excellent) [19]. In addition to the ICC, kappa statistic was also computed to assess the extent of agreement between the subsequent administrations of AREALD-30 and AREALD-99. (Kappa agreements; <0.20-poor; 0.21–0.40-fair; 0.41–0.60-moderate; 0.61–0.80-substantial; 0.81–1.00-almost perfect) [20]. To assess validity of our instrument, convergent, discriminant, predictive and construct validity tests were performed. For convergent validity, Spearman correlations were calculated between AREALD-30 and AREALD-99. The distribution of the AREALD-30 across different educational levels was tested to explore discriminant validity and confirm differences, through a nonparametric test (Kruskal-Wallis). For predictive validity, correlation of AREALD-30 with self-perceived oral health status, dental visiting habits and A-OHIP-14 were calculated. To assess the construct validity of the AREALD-30 based on a conceptual model, Confirmatory Factor Analysis (CFA) was conducted. CFA evaluated the construct validity and the dimensionality of the AREALD-30. The method selected for CFA model estimation was ‘weighted least squares’ with asymptomatic covariance matrix due to the ordinal nature of the data. The fit of the model to the data was assessed using the following indices: Chi-squared goodness of fit statistic, Comparative Fit Index (CFI; ranges from 0 to 1 with values >0.90 acceptable), Non-Normed Fit Index (NNFI; ranges from 0 to 1 with values >0.90 acceptable), Root Mean-Squared Error of Approximation (RMSEA ranges from 0 to 1 with values <0.08 acceptable), Standardized Root Mean-square Residual (SRMR ranges from 0 to 1 with values <0.08 acceptable) and Parsimonious Normed Fit Index (PNFI) [21]. While most of the traditional psychometric analyses focus on an instrument’s total score, Item Response Theory (IRT) models consider each item of a given instrument as unique trait [22]. Since the original version of the REALD-30 was designed to have one dimension, the unidimensionality of the AREALD-30 was also evaluated in a Rasch analysis using the Partial Credit Model [23]. The Rasch analysis approach has been described in detail elsewhere [12]. The rating scale instrument quality must include the following if the rating is to be good; 1) Item model fit, mean range square range extremes between 0.5 and 2.0, 2) person and item reliability estimates greater than 0.81, 3) person separation between 3.0 and 4.0, 4) less than 2% of scores not maximum extreme or minimum extreme (all subjects getting the question right or wrong), and 5) percent of variance in data explained by measures should be between 60% and 70% [24]. In addition, the item quality was evaluated by determining if all items correlated positively with the total score. Data were analyzed using the Winsteps program version 3.61.2 (Winsteps, Chicago, IL, USA) as well as LISREL 8.80.

## Results

Most of the participants were young adults and the mean age of the study population was 28.7 years. Table 1 demonstrates that there were more male participants than females and a majority of the subjects were University graduates. Approximately, one third (32.8%) of the study population had never been to a dentist. Poor and excellent ratings of self-perceived oral health status were provided by few participants and most of the subjects recorded fair to very good rating.

**Table 1:** **Background characteristics of the study population (n = 177)**

Characteristic	n (%)	AREALD-30	AREALD-99
		Mean (SD)	Mean (SD)
**Gender**			
Males	110 (62.1%)	21.6 (6.9)	74.1 (20.3)
Females	67 (37.9%)	22.5 (6.8)	76.4 (20.6)
**Educational status**			
Primary	6 (3.4%)	15.5 (10.1)	55.2 (34.2)
Intermediate	9 (5.1%)	19.3 (7.3)	69.7 (21.6)
Secondary	44 (24.9%)	21.5 (4.8)	72.9 (15.9)
Graduation	107 (60.5%)	22.7 (6.5)	77.9 (18.8)
Post-graduation	11 (6.2%)	23.3 (4.6)	78.3 (13.3)
**Dental visit**			
Visited within previous 6 months	46 (26.0%)	22.6(5.7)	76.5 (16.1)
Visited within previous 6 -12 months	73 (41.2%)	22.8 (6.6)	78.5 (18.1)
Never been to dentist	58 (32.8%)	20.2 (7.6)	69.0 (24.5)
**Self-perception of oral health status**			
Poor	17 (9.6%)	17.8 (8.4)	63.0 (28.4)
Fair	59 (33.3%)	21.9 (7.4)	75.9 (20.7)
Good	47 (26.6%)	22.3(5.6)	75.1 (16.7)
Very good	38 (21.5%)	22.7(6.5)	76.4 (19.1)
Excellent	16 (9.0%)	23.8 (6.0)	80.0 (19.8)

*n - number of participants.*

### Reliability

The internal consistency of both the Arabic word recognition instruments was good, Cronbach’s alpha was found to be 0.89 and 0.91 for AREALD-30 and AREALD-99 respectively. The ICC used to examine the test-retest reliability was higher than 0.90 for all the instruments, indicating that there was an excellent agreement between the repeated administrations (Table 2).

**Table 2:** **Descriptive statistics and reliability of AREALD-30 and AREALD-99**

	Mean	SD	Minimum	Maximum	Cohen kappa	ICC (95% CI)	Cronbach's alpha
**AREALD-30**	21.97	6.85	0	30	0.83	0.992 (0.979-0.997)	0.89
**AREALD-99**	74.94	20.37	5	99	0.81	1.00 (0.999-1.00)	0.91

### Validity

AREALD-30 correlated significantly and positively with the other oral health literacy tool, AREALD-99 (Table 3). However, AREALD-30 did not correlate significantly with A-OHIP-14, self-perceived oral health status and dental visiting habits. There were significant differences in AREALD-30 across categories of educational levels of the subjects (p = 0.02). Higher scores on the AREALD-30 were seen in adults with higher educational status (Table 1). AREALD-30 was tested for the original one factor structure (Model 1) using CFA and the results indicated that the fit indices did not meet the criteria of acceptable model fit. According to the LISREL output, some modifications were required to improve. The two factor model (Model 2) demonstrated a better fit than did the Model 1 (χ2 = 1803.87, df = 405). Other fit indices indicated better fit as well (CFI = 0.89, NNFI = 0.88, PNFI = 0.79, RMSEA =0.14).

**Table 3:** **Spearman correlation coefficients of AREALD-30 with AREALD-99, A-OHIP-14, self-perceived oral health status and dental visiting habits**

	AREALD-99	A-OHIP-14	Self-perceived oral health status	Dental visiting habits
**AREALD-30**	0.959*	-0.105	0.136	-0.142

*p < 0.01.

**Figure 1:**
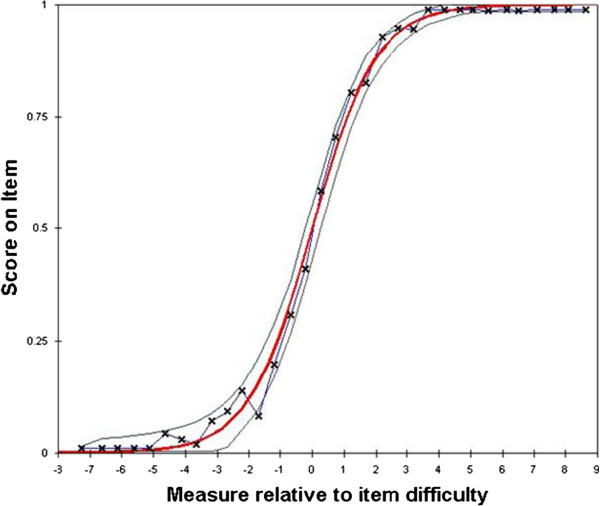
**Average item characteristic curve depicting empirical data to mathematical model fit.** It shows the probability of subjects, with differing ability, scoring correctly (a score of 1) on an average item.

Rasch analysis of AREALD-30 is presented in Table 4. The in-fit mean-square statistics for AREALD-30 were all within the desired range of 0.50 - 2.0. As the outfit mean-square statistics are more sensitive to outliers, some items were outside the range (gingiva, sugar, smoking, floss, extraction and brush). The person and item reliability estimates (Cronbach’s alpha) were 0.86 and 0.98 respectively; easily meeting the desired amounts. The person separation index was 2.45 with extremes and 2.80 without extremes; almost meeting the desired 3.0. Twenty-two participants achieved a maximum score (12.4%) and one participant received a minimum score (0.6%). The amount of variance explained by Rasch measures was 50.9%. Finally, all items correlated positively with the estimated measure. The average correlation was 0.53 (SD = 0.11), with a range of 0.25 to 0.66. Figure 1 demonstrates the empirical data to mathematical model fit by plotting model, data, and 95% confidence intervals around the measure. The vertical axis is the expected score on the average item plotted against the horizontal axis which is the Rasch estimate of dental literacy. The thick curved line is the Rasch mathematical model, the thinner lines on either side are the 95% confidence interval of the model, and the ‘x’s joined by the jagged line are the empirical data. The tight agreement between the actual data and the mathematical model suggests good data to model fit. The mathematical model explains 50.9% of the variance in the observations, with differing knowledge of the participants explaining 21.3% and the differing difficulty of the items explaining 29.6%. The exceptions being in the extreme lower end of the scale and the extreme higher end of the scale where the 95% confidence intervals suggest less accuracy in the estimates.

**Table 4:** **Rasch analysis of AREALD-30**

Items	Item mean	Infit MNSQ	Infit ZSTD	Outfit MNSQ	Outfit ZSTD
Temporomandibular	0.34	1.08	0.5	1.34	0.6
Hypoplasia	0.36	0.78	-1.4	0.50	-0.2
Plaque	0.42	0.85	-1.0	1.37	0.6
Braces	0.49	1.37	2.30	1.52	0.80
Cellulitis	0.45	0.85	-1.0	0.82	0.0
Apicoectomy	0.49	1.18	1.0	1.14	0.4
Fluoride	0.63	0.74	-1.60	0.54	-1.0
Bruxism	0.66	0.71	-1.8	0.77	-0.3
Pulp	0.65	0.83	-0.9	1.36	0.8
Periodontal	0.62	0.86	-0.7	0.61	-0.8
Enamel	0.60	0.92	-0.3	0.71	-0.5
Restoration	0.71	0.64	-2.1	0.47	-1.3
Fistula	0.63	1.44	2.0	1.42	0.9
Sealant	0.72	1.25	1.2	1.20	0.5
Genetics	0.82	1.10	0.5	1.25	0.6
Incipient	0.81	0.76	-1.1	0.50	-0.9
Dentition	0.81	0.96	0.0	0.55	-0.7
Abscess	0.82	1.09	0.4	1.20	0.5
Malocclusion	0.80	1.13	0.5	0.62	-0.3
Denture	0.89	0.68	-1.2	0.36	-0.6
Gingiva	0.91	1.28	1.0	7.75	3.4
Hyperemia	0.87	0.69	-1.1	0.51	-0.2
Analgesia	0.84	1.11	0.4	0.73	0.1
Sugar	0.93	0.66	-1.0	0.22	-0.4
Smoking	0.94	0.93	0.0	0.33	0.0
Floss	0.93	1.15	0.4	5.25	1.8
Extraction	0.93	1.23	0.6	7.56	2.0
Halitosis	0.96	0.91	0.0	0.20	0.1
Caries	0.95	0.72	-0.4	0.27	0.5
Brush	0.98	1.16	0.4	9.00	2.7

## Discussion

Currently, there are no oral health literacy instruments available for use in the Arabic speaking gulf peninsula. To our knowledge, this is the first study that has attempted to introduce and evaluate the psychometric properties of an oral health literacy instrument for Arabic speaking population. AREALD-30 demonstrated excellent internal consistency and reliability on repeated administrations. It was also significantly related to AREALD-99 and educational status, therefore exhibited good convergent and concurrent validity.

Oral health literacy may be a determinant to oral health [25]. Therefore, there is a need to identify individuals with low oral health literacy in every population which requires appropriate oral health literacy instruments. Although word recognition instruments are not comprehensive and ideal, they are instruments of choice in patient care settings as they are easy to administer and take very less time. Widely used word recognition instruments in the field of dental health literacy are REALD-30 and REALD-99. Although both the instruments have good internal reliability and construct validity [6], we have preferred REALD-30 for Arabic adaptation over REALD-99 as it is less time consuming and causes less burden to the respondent. The proponents of REALD have also recommended use of REALD-30. As there are no validated word recognition instruments in Arabic, we have also translated REALD-99 into Arabic only to evaluate the convergent validity of AREALD-30.

The internal consistency expressed as Cronbach α of both AREALD-30 and AREALD-99 was found to be excellent. These findings are consistent with those from previous studies on REALD [6, 7] and Hong Kong Rapid Estimate of Adult Literacy in Dentistry (HKREALD-30) [12]. For evaluating temporal stability, we have also assessed the test-retest reliability which was found to be excellent for both AREALD-30 and AREALD-99. AREALD-30 exhibited good convergent validity and had an excellent correlation with AREALD-99. However, AREALD-30 was limited in terms of predictive validity and could not relate to A-OHIP-14, self-perceived dental health status or dental visiting habits, which are a few known proxy measures of clinical oral health status. The probable reason for no correlations existing between AREALD-30 and the oral health outcomes might be due to the lack of communicative and critical health literacy components in a word recognition instrument like AREALD-30 which also can influence final health outcomes [26]. Moreover, word recognition instruments might not be capable to capture the functional literacy to its fullest [8] which is also related to the health outcomes [26]. In contrast, English versions, REALD-30 [7] and REALD-99 [6], were significantly related to OHIP-14. AREALD-30 exhibited good concurrent validity with better literacy scores being reported by subjects with greater educational attainment and vice-versa. In congruence with this study, data from nationally representative sample of the United States also reports that lower educational attainment is associated with lower estimated health literacy [27].

The CFA showed presence of two factors in agreement with the original REALD-30 [7]. We have also conducted the Rasch analysis as it measures a person’s ability and the difficulty of each questionnaire independently, along the common measurement continuums [28]. In addition, it was an acceptable fit for our purposes as we were not interested in separating out the top few participants. Further, the Rasch analysis supports the use of the items in their current form as they all contribute to the measure, and all are measuring a different attribute of literacy; as evidenced by appropriate mean-square estimates [29–31]. As the outfit mean-square statistics are more sensitive to outliers, some items were outside the range (Gingiva, Sugar, Smoking, Floss, Extraction and Brush) in Rasch analysis. Misfit of items indicates a lack of the expected probabilistic relationship between the item and other items in the scale. This introduces noise into the measurement, diminishing the instrument’s quality. Misfitting items are usually removed until there is no further improvement in the fit requirements [28, 32]. However, before considering removing of these words, further studies on larger populations are required to observe the validity of these findings. In addition, as we are not concerned with the extreme measures and outliers, the Infit mean-square statistics are most useful to our analysis. The items were not removed as the Infit mean-square statistics were acceptable.

Twenty-two participants achieved a maximum score (12.4%) and one participant received a minimum score (0.6%). As we were not concerned about the subjects with highest scores, the percentage of subjects with extreme measures was not a deterrent to the use of AREALD-30. The instrument performed well from the lower to upper ranges - the area of most interest. The amount of variance explained by Rasch measures was 50.9%, which is quite acceptable for an instrument with no high stakes. Finally, all items correlated positively with the estimated measure and exhibited a good model fit which supports the use of the AREALD-30 in measuring oral health literacy.

However the study had a few limitations; it was constrained by a small sample size that was recruited by a non-probability sampling procedure from a dental clinic environment which reduces the generalizability of the study findings. Also, it did not evaluate the oral clinical status of the subjects, which is an ideal outcome measure that could be helpful in assessing the predictive validity of the AREALD-30.

## Conclusions

The AREALD-30 showed excellent reliability on repeated administrations and demonstrated very good internal consistency. Although, AREALD-30 exhibited good convergent and concurrent validity, its predictive validity was poor. The Rasch analysis supported the use of AREALD-30, by extending the classical test theory information from, beyond the fit of the mean to the fit of each of the items in the instrument to each of the subjects. Each of the items demonstrated to have good fit to the data, and the subjects of concern were demonstrated to fit the model. Further studies on larger sample sizes selected from a diverse population are recommended to assess the generalizability of AREALD-30. It would also be interesting to see the responsiveness and sensitivity of the instrument to change across time.
